# Anisotropic electronic conduction in stacked two-dimensional titanium carbide

**DOI:** 10.1038/srep16329

**Published:** 2015-11-09

**Authors:** Tao Hu, Hui Zhang, Jiemin Wang, Zhaojin Li, Minmin Hu, Jun Tan, Pengxiang Hou, Feng Li, Xiaohui Wang

**Affiliations:** 1 Shenyang National Laboratory for Materials Science, Institute of Metal Research, Chinese Academy of Sciences, 72 Wenhua Road, Shenyang 110016, China; 2 University of Chinese Academy of Sciences, Beijing 100049, China

## Abstract

Stacked two-dimensional titanium carbide is an emerging conductive material for electrochemical energy storage which requires an understanding of the intrinsic electronic conduction. Here we report the electronic conduction properties of stacked Ti_3_C_2_*T*_2_ (*T* = OH, O, F) with two distinct stacking sequences (Bernal and simple hexagonal). On the basis of first-principles calculations and energy band theory analysis, both stacking sequences give rise to metallic conduction with Ti 3*d* electrons contributing most to the conduction. The conduction is also significantly anisotropic due to the fact that the effective masses of carriers including electrons and holes are remarkably direction-dependent. Such an anisotropic electronic conduction is evidenced by the *I*−*V* curves of an individual Ti_3_C_2_*T*_2_ particulate, which demonstrates that the in-plane electrical conduction is at least one order of magnitude higher than that vertical to the basal plane.

Typical two-dimensional (2D) materials like graphene^1^ and inorganic graphene analogues^2^ (IGAs) exhibit unique properties for sensing^3^, catalysis^4^, and energy storage applications^5^. Recently, a new family of IGAs called MXene^6^ has been successfully synthesized by chemically etching the layered ternary carbides/nitrides (referred to as MAX phases^7^, which have weak coupling between MX layers juxtaposed with strong in-plane bonds^8^). For instance, the first reported MXene, Ti_3_C_2_*T*_2_ (*T* = OH, O, F) was obtained by selectively etching off the Al layer from Ti_3_AlC_2_ with hydrofluoric acid. Simultaneously, the chemically active surface of the remained Ti_3_C_2_ blocks are spontaneously decorated with primary OH, and a few O and F. Like many 2D materials that exist in bulk form as stacks of strongly bonded layers with weak interlayer attraction^9^,^10^,^11^,^12^, MXene sheet also tends to restack. Very recently, the stacked form of MXene, Ti_3_C_2_*T*_2_ ‘clay’ and its analogues were reported to have ultrahigh volumetric capacitances of up to 900 F cm^−3^ for energy storage^13^,^14^,^15^,^16^,^17^. These pioneering works on precious-metal-free materials for electrochemical energy storage have attracted wide scientific attentions. In such an electron-involved application, it is crucial to know the electronic conduction properties of the materials. However, the intrinsic electronic conduction properties of the amazing Ti_3_C_2_*T*_2_ along and perpendicular to the basal plane remain unknown.

Here, we report, for the first time, on the intrinsic electronic conduction properties of stacked Ti_3_C_2_(OH)_2_ by both theoretical prediction and *in situ* experimental measurements. In this work, two distinct stacked structures and their corresponding electronic structures were systematically investigated by dispersion-corrected density functional theory (DFT-D), in which long-range dispersion interactions are taken into consideration (its validity was tested on graphite and MoS_2_, see Supplementary Table S1). The origin of the electronic conduction and conduction anisotropy is revealed. Remarkably, the *I−V* curve measurements excellently verify our theoretical prediction.

## Results

### Crystal structure

**Figure 1:**
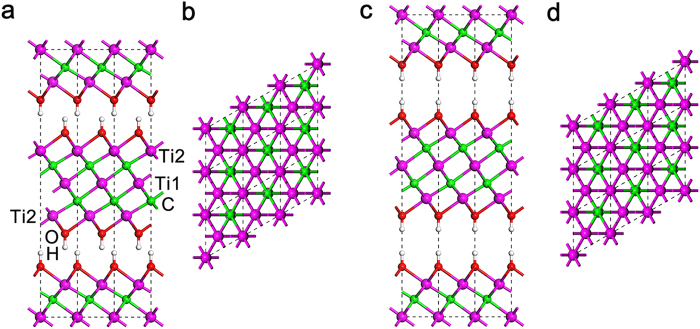
Projections of two distinct Ti_3_C_2_(OH)_2_ stacking types. Side- and top- view of (**a,b**) Bernal and (**c,d**) SH Ti_3_C_2_(OH)_2_. A 3 × 3 supercell is used in each projection.

The performance of Ti_3_C_2_*T*_2_ in practical applications is believed to be dominated by its stacking manner. Nevertheless, most theoretical works focused on monolayer MXene^18–20^ instead of their stacked forms. In this study, the stacked Ti_3_C_2_*T*_2_ was systematically investigated by DFT-D in which the long-range interactions are taken into accounts. Since the termination is primarily OH functional group^13^,^21^, Ti_3_C_2_*T*_2_ in this work is simplified to Ti_3_C_2_(OH)_2_ (see Supplementary Table S2 for detailed structure information). According to the relative position of adjacent layers, there are two distinct stacking types^21^,^22^,^23^,^24^ for Ti_3_C_2_(OH)_2_, *i.e.*, *AA* (simple hexagonal, SH) and *AB* (Bernal). Figure 1 presents the projection of the two stacking configurations.

The formation energies of the two distinct stacking configurations were calculated with two schemes of PW91-OBS (refs 25,26) and PBE-Grimme (refs 27,28). The results are summarized in Table 1. From an energetic point of view, the formation energies of Bernal and SH Ti_3_C_2_(OH)_2_ are all positive, implying that both of them are thermodynamically stable. The Bernal stacking is somewhat more energetically favorable than the SH one. It is noted that the energy difference between the two stackings is quite small (<65 meV/atom), which is consistent with the previous calculation results^21^.

### Valence charge density distribution

**Figure 2:**
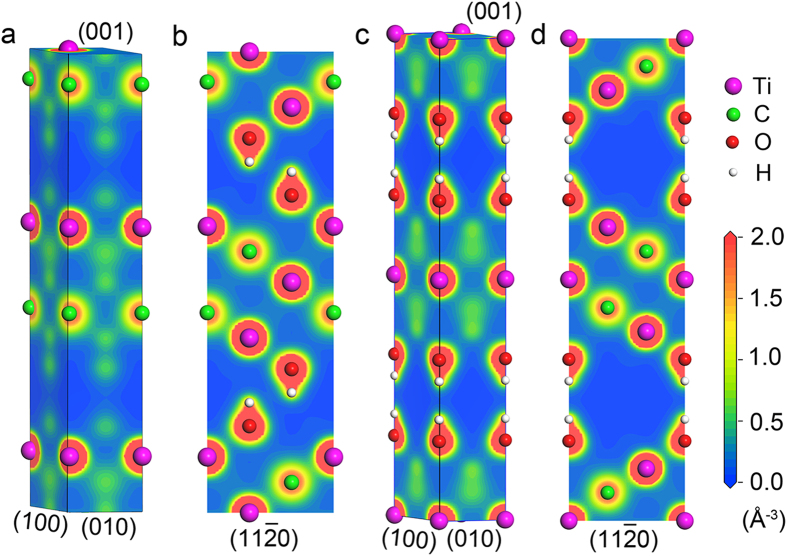
Electron density distribution in low-index planes. (**a,b**) Bernal and (**c,d**) SH Ti_3_C_2_(OH)_2_. Note that an interlayer electron density dilution zone exists in both stacking types.

Valence electrons contribute to chemical bonding and most electronic conduction^29^. The electronic structure of stacked Ti_3_C_2_(OH)_2_ in real space was investigated by valence electron density based on the optimized crystal structures. Figure 2a–d show the distribution of the valence electron density on specific planes of the stacked Ti_3_C_2_(OH)_2_. For both stacked structures, they share a common feature that the intralayer bonding is strong, while the interlayer is weak since there are electron density dilution zones between neighboring Ti_3_C_2_(OH)_2_ layers. The strong intralayer bonding is inherited from the Ti_3_C_2_ blocking sheet of Ti_3_AlC_2_ in which *pd* hybridization or *pd* bonding^8^ of Ti 3*d*−C 2*p* predominates. The covalently bonded chain Ti2–C–Ti1–C–Ti2 (Fig. 2) is reserved by the OH group, which forms bonds with surface Ti and C atoms. The weak interlayer bonding is derived from the long-range electrostatic interaction between the adjacent Ti_3_C_2_(OH)_2_ layers. The interaction is dominated by surface OH terminations perpendicular to the layers. The existence of interlayer electron density dilution zone in both structures suggests that few if any electrons can be transferred directly between the layers.

### Band structure

**Figure 3:**
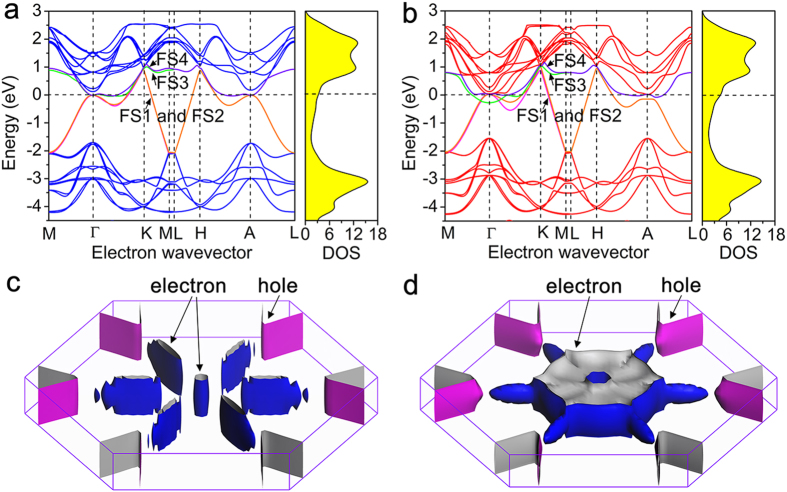
Band structure and Fermi surface. (**a,b**) Band structure and (**c,d**) corresponding FS of (**a,c**) Bernal and (**b,d**) SH Ti_3_C_2_(OH)_2_. FS1 (magenta) and FS2 (orange) are partially degenerated. FS3 (green) and FS4 (violet) are partially degenerated. Note that these weakly dispersive bands are responsible for the formation of electron and hole pockets.

Band structure describes the relation between electronic energy and electron wavevector in momentum space. The microscopic behavior of electrons in a solid is most conveniently specified in terms of the electronic band structure^30^. Figure 3a,b depict the band structures and density of states (DOS) in specific directions of the Brillouin zone (as shown in Supplementary Fig. S1). For the sake of comparison, we chose two mirror paths (M−Γ−K−M and L−H−A−L) for each structure. There are three main features for the band structures: First, they are both dominated by 2D in-plane covalent bonds, with the modification of weak interlayer interactions. As a result, the band structure shows a strongly anisotropic feature with less energy dispersion along *c*-axis. As presented in Fig. 3a,b, the dispersion of the bands perpendicular to the basal planes (M−L) is almost negligible, demonstrating essentially a 2D character of the electronic structures. The anisotropy of the band structures near and below the Fermi energy (*E*_*F*_) indicates that the conductivity is also anisotropic. Specifically, the electrical conductivity along the *c*-axis should be much lower than that in the basal plane. This feature can be also found in the free-standing monolayer and bilayer Ti_3_C_2_(OH)_2_ (Fig. S2). Second, resembling that of monolayer^16^,^18^ and bilayer Ti_3_C_2_(OH)_2_, the band structure exhibits typical metallic conduction with bands crossing *E*_*F*_ along various directions, which results in a finite DOS (3.19 states/eV cell and 4.51 states/eV cell for Bernal and SH, respectively) at *E*_*F*_. Third, at *E*_*F*_, the DOS mainly originates from the nearly free electron states^31^ of Ti2 3*d* and Ti1 3*d* (88% of the total DOS at *E*_*F*_ for Bernal and 68% for SH Ti_3_C_2_(OH)_2_, see Supplementary Fig. S3). Since the electronic transport properties are governed by the electrons near *E*_*F*_, the 3*d* electrons of Ti contribute predominantly to the electronic conduction.

### Fermi surface

The band occupation around *E*_*F*_ accounts for the metallic nature of matters, and the electrons at the Fermi surface (FS) determine the transport properties like conductivity of materials^32^. It is thus important to establish the shape of FS. Figure 3c,d illustrate the shape of the whole FS. Corresponding to the fact that four double-degenerate half-filled bands across *E*_*F*_ in the band structure (denoted as FS 1, 2, 3 and 4 in Fig. 3a,b), there are four envelopes consisting of the whole FS. Red regions correspond to pockets of holes and blue regions to pockets of electrons. Both FSs show a hexagonal electron pocket around the c^*^ axis in reciprocal space and surrounding six cylindrical hole pockets. Around c^*^ there is a region of electrons, and a relatively flat band across *E*_*F*_ along Γ–K and H–A. The FS of Bernal is disconnected while that of SH is connected by electron pockets. Interestingly, the hole-like pockets in the FSs of the two configurations are similar: cylindrical hole pockets are around H and K. In consistent with the band structure analysis, the FSs of stacked Ti_3_C_2_(OH)_2_, as well as those of monolayer and bilayer Ti_3_C_2_(OH)_2_ (Supplementary Fig. S4), all show obvious 2D features.

### Effective mass

The effective masses of carriers including electrons and holes represent their response to the applied external fields. Generally speaking, an electron in a periodic potential is accelerated relative to the lattice in an applied electric or magnetic field, as if the mass of the electron is equal to its effective mass^32^. To understand the dynamics of electrons and holes, the effective masses for electron 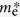 and hole 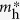 were evaluated along the parabola at several points indicated in Supplementary Figs S5 and S6. Fitting these parabolas can give the minimums for the effective mass since the dispersion is strong in these directions (Fig. 3a,b). The effective masses of carriers at high-symmetry points in Ti_3_C_2_(OH)_2_ are listed in Table 2. The fitted parabolas are shown in Supplementary Figs S7 and S8. It is found that the effective mass in the basal plane are quite small (<0.5 *m*_0_, Table 2), in stark contrast, the effective electron and hole masses perpendicular to the layers are extremely large (they are estimated to be infinite by fitting nearly flat parabolas along Γ–A). Therefore, the carriers respond much more quickly in the basal plane than those along the *c*-axis, which agrees with the afore-discussed features of electron density distribution, band structure and FS shape. In the basal plane, the holes’ effective masses are generally one order of magnitude less than those of electrons, indicating that holes response to applied electric field much more easily.

## Discussion

**Figure 4:**
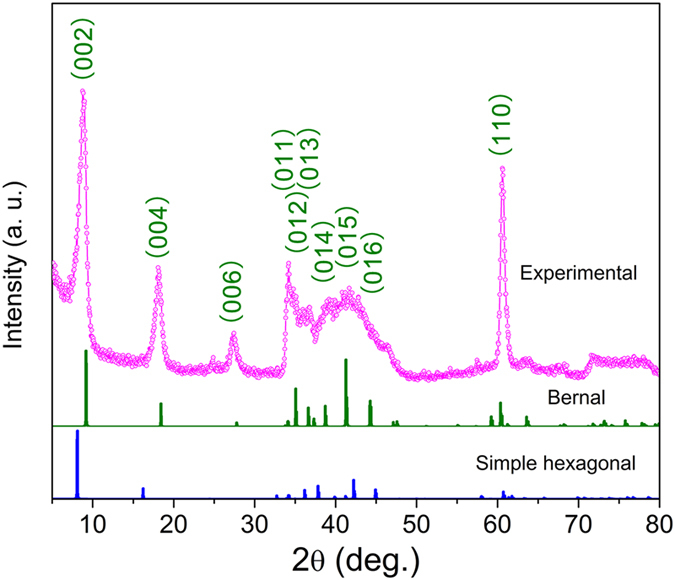
Experimental and simulated XRD patterns.

The present results obtained by *ab initio* calculation indicate that the formation energies of both Bernal and SH Ti_3_C_2_(OH)_2_ are all negative, suggesting that both stacking sequences are thermodynamically stable. Besides, the Bernal Ti_3_C_2_(OH)_2_ is somewhat more energetically favorable than the SH one, and its XRD pattern matches the experimental pattern better than that of SH one (Fig. 4, see Supplementary Table S3 for simulated XRD data). However, this does not necessarily rule out the SH structure because most of its calculated peaks match the experimental XRD pattern well too (Fig. 4). In fact, the selected area electron diffraction shows a feature of non-periodic combination of different stacking along [0001] (see Supplementary Fig. S9). It is believed that both stacking structures likely coexist in the prepared sample. Anyway, as shown in the band structures, no matter which specific structure the stacked Ti_3_C_2_(OH)_2_ has, the electron conduction is highly anisotropic.

The anisotropy is more straightforwardly shown in the FS than the band structures. FS is the boundary between ground states and excited states. The volume inside of the FS corresponds to occupied states while the outside represents unoccupied states at zero temperature. The volume inside the surface determines the low-energy free carrier plasmon frequencies and the electrical conductivity^10^. Since the electron velocities are orthogonal to the FS, the flat part at the top and bottom of the Brillouin zone (Fig. 3d) gives rise to the most important contributions to the conduction along the *c* direction, whereas the transversal part of the FS is mainly responsible for the in-plane conduction. For the two structures, the Fermi cylinder of hole shows obvious features of 2D materials^33^, indicating that the carrier in two structures is transferred preferentially in the basal plane.

**Figure 5:**
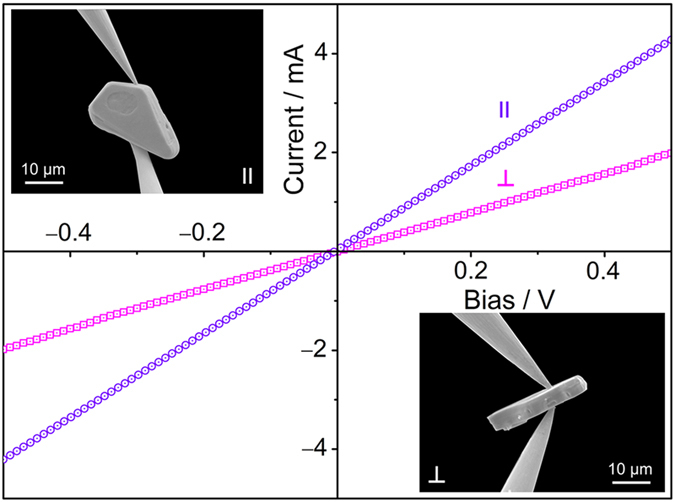
Orientation-dependent *I−V* curves and SEM images of an individual Ti_3_C_2_*T*_2_ particulate. The estimated in-plane electrical conductivity is one order of magnitude higher than the vertical electrical conductivity.

To experimentally testify the predicted conductivity anisotropy, *I*–*V* curves were measured on individual Ti_3_C_2_*T*_2_ particulates with two distinct morphologies, i.e., laminated-structure-free and accordion-like. Ti_3_C_2_*T*_2_ particulates with the two morphologies are both conductivity anisotropic (Fig. 5 and Fig. S10). The estimated in-plane electrical conductivity is one order of magnitude higher than that vertical to the basal plane (see Supplementary Fig. S11 for the discussions on contract resistance). Interestingly, by *in situ I*–*V* measurement on accordion-like Ti_3_C_2_*T*_2_ particulates (Supplementary Fig. S10), it is found that the electronic conductivity vertical to the basal plane is highly mode-dependent (stretching or shrinking). The value measured in shrinking mode is at least 10 times higher than that measured in stretching mode. This mode-sensitive feature can be used to manufacture micro-displacement-sensitive devices.

In summary, *ab initio* calculations have demonstrated that the electronic conduction of both Bernal and SH Ti_3_C_2_(OH)_2_ is highly anisotropic, which was experimentally evidenced by the *in situ I−V* measurements of an individual Ti_3_C_2_*T*_2_ particulate. Quantitatively, the in-plane electrical conductivity is at least one order of magnitude higher than that vertical to the basal plane. The excellent consistency between first-principles prediction and experimental results demonstrated in this work may lead to a comprehensive understanding on the structural and electronic properties of the emerging MXenes in stacked form. Moreover, it offers fundamental information for exploring the electron-involved applications of MXenes.

## Methods

### First-principles calculations

The DFT and DFT-D calculations were performed using the Cambridge Sequential Total Energy Package (CASTEP)^34^,^35^. The electron-ion interaction was represented by using plane-wave pseudo potential. Vanderbilt-type ultrasoft potentials^36^ were utilized for the calculations. Configurations of H−1*s*^1^, C−2*s*^2^2*p*^2^, O−2*s*^2^2*p*^4^, Ti−3*s*^2^3*p*^6^3*d*^2^4*s*^2^ were treated as valence electrons. The electronic exchange correlation energy was treated as GGA-PBE and GGA-PW91. The long-range interaction was considered by dispersion correction^37^ within the OBS and Grimme methods. The Monkhorst-Pack scheme^38^ with 9 × 9 × 2 *k* points meshes were used for the integration in the irreducible Brillouin zone so that the individual spacing was less than 0.05 Å^−1^ (9 × 9 × 1 *k* points meshes for monolayer and bilayer). We performed cutoff energy evaluation on the calculations results. It was found that increasing the energy from 380 to 500 eV within the identical calculation scheme gives rise to negligible changes in total energy, lattice parameter and atomic position. Thereby, the cutoff energy was set as 380 eV. The Broyden–Fletcher–Goldfarb–Shanno minimization scheme^39^ was used to minimize the total energy and interatomic forces. Fermi level was smeared by 0.1 eV. The convergence for energy was chosen as 1.0 × 10^−9^ eV/atom, and the structures were relaxed until the maximum force exerted on the atoms became less than 0.001 eV/Å. Electronic structure calculations were carried out with GGA–PW91–OBS scheme.

### Preparation of Ti_3_C_2_ *T*_2_

Individual Ti_3_C_2_*T*_2_ particulates with two distinct morphologies, i.e., laminated-structure-free and accordion-like were prepared in a mild etching condition and a harsh condition, respectively. For the laminated-structure-free Ti_3_C_2_*T*_2_ particulates, the etching process was carried out by immersing the porous Ti_3_AlC_2_ monolith in a diluted hydrofluoric acid solution (HF, 10 wt.%) for 72 h at room temperature. The porous Ti_3_AlC_2_ was prepared by the method reported previously^40^. After that, the resulting sediment was washed several times with deionized water and immersed therein for one day, followed by vacuum filtration. The filtered sample was subsequently dried overnight at 70 °C in an oven. The accordion-like lamellas for the investigation were synthesized by exfoliating porous Ti_3_AlC_2_ in HF (40 wt.%) for 24 h. After that, the resulting sediment was washed several times with deionized water and immersed therein for one day, followed by vacuum filtration. The filtered sample was subsequently dried by supercritical carbon dioxide drying under a condition above the critical point of CO_2_ (*T*_C_ = 31.1 °C, *P*_C_ = 7.39 MPa). The filtered sample was transferred into an autoclave in which ethanol was filled to minimize the evaporation of water from the wet sample during the following processes of heating and pressurizing. As soon as the autoclave was heated up to 40 °C, CO_2_ pre-heated to that temperature was pumped into the autoclave at a flow rate of 0.54 kg h^−1^ to a pressure of 100 bar. After the solvent was completely replaced at that pressure by CO_2_ for 5 h, the CO_2_ was vented by slowly reducing the pressure to ambient (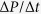 = −3 bar min^−1^) while keeping the temperature.

### Focused ion beam for TEM specimen preparation

The specimens for TEM observations were prepared on a Helios Nanolab 650 dual-beam SEM/FIB system equipped with Nova nanoSEM 430 (FEI, Oregon, USA).

### TEM observation

The microstructural characterizations were performed using a transmission electron microscope (FEI Tecnai G2 F20, Oregon, USA) equipped with a high-angle annular dark-field detector in the scanning transmission electron microscopy system.

### XRD determination

The as-synthesized Ti_3_C_2_*T*_2_ samples were examined by X-ray diffraction (XRD) (Rigaku D/max-2400, Tokyo, Japan) with Cu Kα radiation (λ = 1.54178 Å) at a scanning speed of 0.04° per step.

### *I**−**V* curve measurement

The measurements were conducted in a field-emission scanning electron microscope (FEI, NanoSEM 430) equipped with a four-probe micromanipulator (Kleindiek MM3A-EM) in its vacuum chamber at room temperature. The silicon wafer was used as a substrate to support the well distributed MXene particulates, which were ultrasonically dispersed in an ethanol solution prior to dropping them onto the substrate. For picking up the particulate and measuring its *I−V* curves, two probes were used to connect to a Keithley 4200–SCS semiconductor characterization system. We employed a commercial tungsten probe due to its sufficient hardness and sharpness to reach the experimental goal. Prior to loading the particulate, two tungsten probes were manipulated to achieve tip-to-tip contact (Fig. 5) and then subjected to Joule heating by applying a scanned voltage from 0 to 10 V several times in order to fully remove the surface tungsten oxide layer on the probes. The final electrical resistance of the treated tungsten probes was measured to be around 7 Ω (Supplementary Fig. S12). The targeted single particulate on the substrate was picked up by manipulating the two probes. Before recording the *I−V* signals, two important steps are necessary: (i) the separation of the picked up particulate from the substrate so that the possible influence of the substrate on the measurements can be ruled out; (ii) the blocking of the electron beam to exclude a possible influence of electron bombardment and charging effects.

## Additional Information

**How to cite this article**: Hu, T. *et al.* Anisotropic electronic conduction in stacked two-dimensional titanium carbide. *Sci. Rep.* **5**, 16329; doi: 10.1038/srep16329 (2015).

## Supplementary Material

Supplementary Information

## Figures and Tables

**Table 1:** Summary of the calculated results for two configurations with two calculation schemes.

Scheme	Configuration	E_total_ (eV)	E_monolayer_ (eV)	E_formation_ (eV)	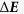 (meV/atom)
PW91-OBS	Bernal	−12078.00	−6036.04	5.92	0
	SH	−12076.89	−6036.04	4.81	61.67
PBE-Grimme	Bernal	−12067.90	−6032.01	3.88	0
	SH	−12067.58	−6032.01	3.56	17.78

*E*_total_ is the total energy of stacked Ti_3_C_2_(OH)_2_. *E*_monolayer_ is the total energy of monolayer Ti_3_C_2_(OH)_2_. *E*_formation_ = 2*E*_monolayer_ − *E*_total_. 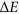 is the energy difference compared with the most stable configuration.

**Table 2:** Effective masses of carriers at high-symmetry points in Bernal and SH Ti_3_C_2_(OH)_2_.

Configuration	FS	Γ	Λ	K	H	Q	R
Bernal	FS1	—	—	−0.0052	−0.0068	—	—
	FS2	—	—	−0.0056	−0.0068	—	—
	FS3	0.1142	0.3324	—	—	0.4658	0.1124
	FS4	0.1090	0.4948	—	—	0.4658	0.1124
SH	FS1	—	—	−0.0072	−0.0063	—	—
	FS2	—	—	−0.0052	−0.0063	—	—
	FS3	0.2845	0.2056	—	—	0.3534	0.1238
	FS4	—	0.3432	—	—	0.3549	0.1236

Unit: *m*_0_. *m*_0_ is the electron rest mass.
