# Supplementary material for: Anisotropic electronic conduction in stacked two-dimensional titanium carbide
Source: Sci Rep. 2015 Nov 9;5:16329. doi: 10.1038/srep16329 (PMC4637832; doi:10.1038/srep16329)
Supplement: Supplementary Information [file srep16329-s1.pdf]

Electronic supplementary information for "Anisotropic electronic conduction in stacked two-dimensional titanium carbide"

Tao Hu[1,2], Hui Zhang[1,2], Jiemin Wang[1], Zhaojin Li[1,2], Minmin Hu[1,2], Jun Tan[1], Pengxiang Hou[1], Feng Li[1] & Xiaohui Wang[1*]

[1]Shenyang National Laboratory for Materials Science, Institute of Metal Research, Chinese Academy of Sciences, 72 Wenhua Road, Shenyang 110016, China, [2]University of Chinese Academy of Sciences, Beijing 100049, China.

Table S1. Calculated lattice constants (in Å) of graphite and MoS_2 with DFT and DFT-D

	Graphite				MoS_2			
Method	DFT	DFT-D		Exp.[1]	DFT	DFT-D		Exp.[2]
Functional	PBE	Grimme	OBS		PBE	Grimme	OBS	
a	2.47	2.46	2.46	2.46	3.18	3.19	3.18	3.16
c	11.74	6.43	6.75	6.70	15.40	12.46	12.74	12.30
d	5.87	3.22	3.33	3.35	4.59	3.13	3.25	3.08

Table S2. Optimized structural data for stacked $Ti_3C_2(OH)_2$

Formula	$Ti_3C_2(OH)_2$	
	Bernal	SH
Space group	$P6_3mc$ (186)	$P6_3/mmc$ (194)
a (Å)	3.06	3.05
c (Å)	19.28	21.89
Atomic positions	Ti1(2/3,1/3,0)	Ti1(0,0,0)
	Ti2(0,0,0.1211)	Ti2(1/3,2/3,0.1081)
	C(1/3,2/3,0.0662)	C(2/3,1/3,0.0584)
	O(2/3,1/3,0.1882)	O(0,0,0.1654)
	H(2/3,1/3,0.2391)	H(0,0,0.2103)

Table S3. Calculated X-ray diffraction data of Bernal $Ti_3C_2(OH)_2$ and SH $Ti_3C_2(OH)_2$ with Cu Kα radiation (λ_1 = 1.54056 Å, λ_2 = 1.54439 Å)

Bernal $Ti_3C_2(OH)_2$				SH $Ti_3C_2(OH)_2$			
(*hkl*)	d_{hkl}	2θ (°)	I/Imax	(*hkl*)	d_{hkl}	2θ (°)	I/Imax
002	9.64	9.17	100.00	002	10.95	8.07	100.00
002	9.64	9.19	50.00	002	10.95	8.09	50.00
004	4.82	18.39	24.61	004	5.47	16.18	13.51
004	4.82	18.44	12.30	004	5.47	16.22	6.76
006	3.21	27.74	5.36	006	3.65	24.37	0.25
006	3.21	27.81	2.68	006	3.65	24.43	0.12
100	2.65	33.77	1.30	008	2.74	32.69	4.11
100	2.65	33.86	0.65	008	2.74	32.78	2.06
101	2.63	34.10	11.67	100	2.64	33.92	1.47
101	2.63	34.19	5.83	100	2.64	34.01	0.73
102	2.56	35.07	47.74	101	2.62	34.17	8.38
102	2.56	35.16	23.87	101	2.62	34.26	4.19
103	2.45	36.63	23.31	102	2.57	34.92	0.41
103	2.45	36.72	11.65	102	2.57	35.01	0.21
008	2.41	37.28	15.16	103	2.48	36.14	11.50
008	2.41	37.38	7.58	103	2.48	36.24	5.75
104	2.32	38.72	25.45	104	2.38	37.80	16.91
104	2.32	38.82	12.72	104	2.38	37.89	8.45
105	2.18	41.29	99.43	105	2.26	39.83	3.74
105	2.18	41.39	49.72	105	2.26	39.94	1.87
106	2.04	44.25	52.61	0010	2.19	41.20	3.73
106	2.04	44.37	26.30	0010	2.19	41.30	1.87
0010	1.93	47.10	6.08	106	2.14	42.21	28.20
0010	1.93	47.23	3.04	106	2.14	42.32	14.10
107	1.91	47.56	7.60	107	2.02	44.89	15.10
107	1.91	47.69	3.80	107	2.02	45.00	7.55
108	1.78	51.18	0.92	108	1.90	47.83	0.16
108	1.78	51.32	0.46	108	1.90	47.95	0.08
109	1.67	55.07	2.32	0012	1.82	49.95	0.37
109	1.67	55.22	1.16	0012	1.82	50.08	0.18
0012	1.61	57.30	0.71	109	1.79	51.00	0.15
0012	1.61	57.46	0.35	109	1.79	51.14	0.76

Table S4. Estimated electrical conductivity of $Ti_3C_2T_2$ by Ohm's law

	R (Ω)	S (μm^2)	l (μm)	ρ (10^{-6} Ω·m)	σ (S m^{-1})
Horizontal	123.5	47	14	415	2.4×10^3
Vertical	275.6	132	5	7276	137

$R = \rho l/S$, $\rho = 1/\sigma$. R is the electrical resistance (obtained from the slope of the I–V curve, and the electrical resistivity of tungsten probes is subtracted). S, l, ρ and σ is the cross-section area, length, electrical resistivity and conductivity of the conductor, respectively.

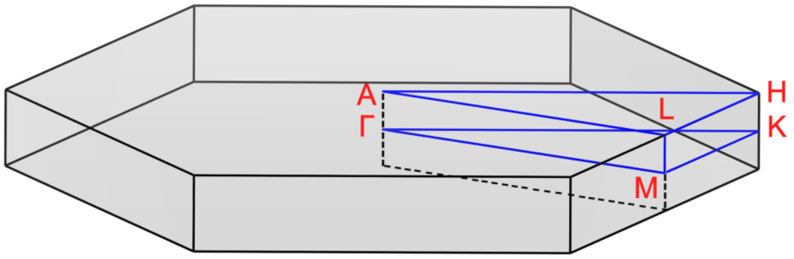

Figure S1. The first Brillouin zone used in this work.

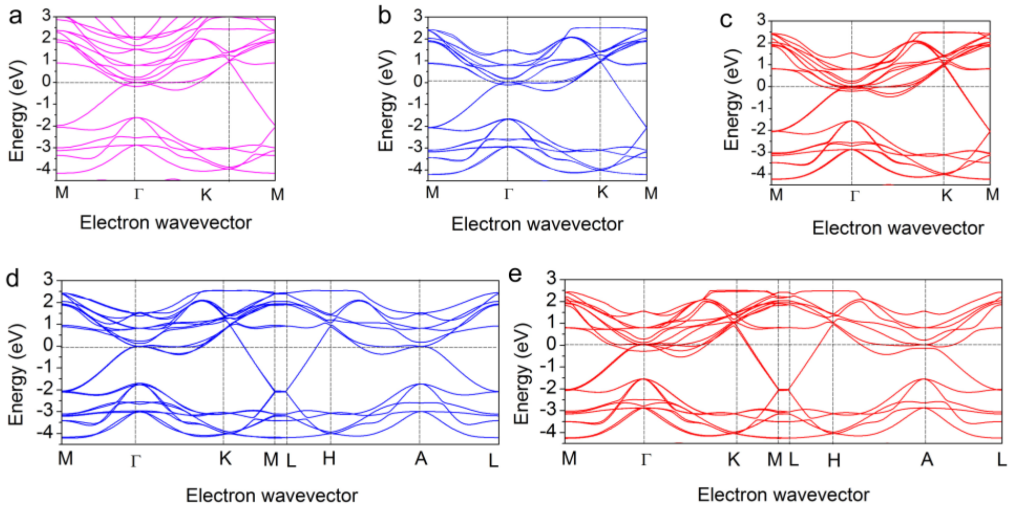

Figure S2. Electronic band structure. (**a**) monolayer, (**b**) Bernal bilayer, (**c**) SH bilayer and (**d**) Bernal multilayer and (**e**) SH multilayer $Ti_3C_2(OH)_2$. Note that the upper band are compressed in stacked $Ti_3C_2(OH)_2$ compared to monolayer.

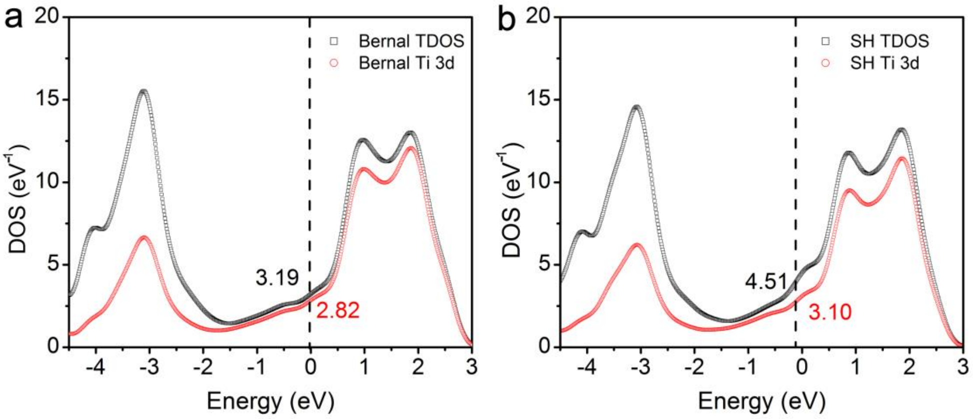

Figure S3. PDOS of Ti 3*d* electrons and TDOS of (a) Bernal and (b) SH $Ti_3C_2(OH)_2$. Near the Fermi level, the DOS mainly originates from nearly free electron states of Ti2 3*d* and Ti1 3*d*. The Ti 3*d* electrons contribute most, 88% for Bernal and 68% for SH $Ti_3C_2(OH)_2$, of the electronic conduction.

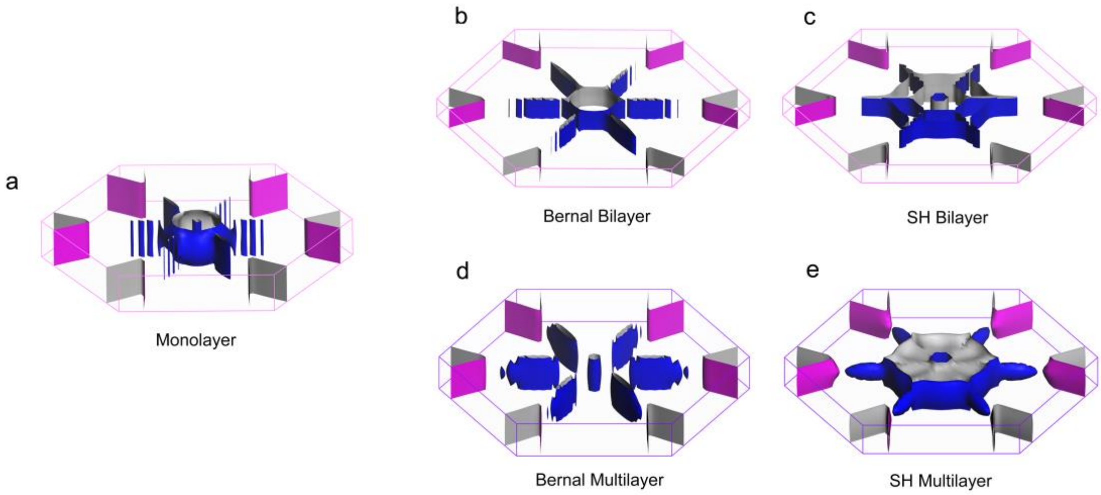

Figure S4. Fermi surface of $Ti_3C_2(OH)_2$. (**a**) monolayer, (**b**) Bernal bilayer, (**c**) SH bilayer and (**d**) Bernal multilayer and (**e**) SH multilayer.

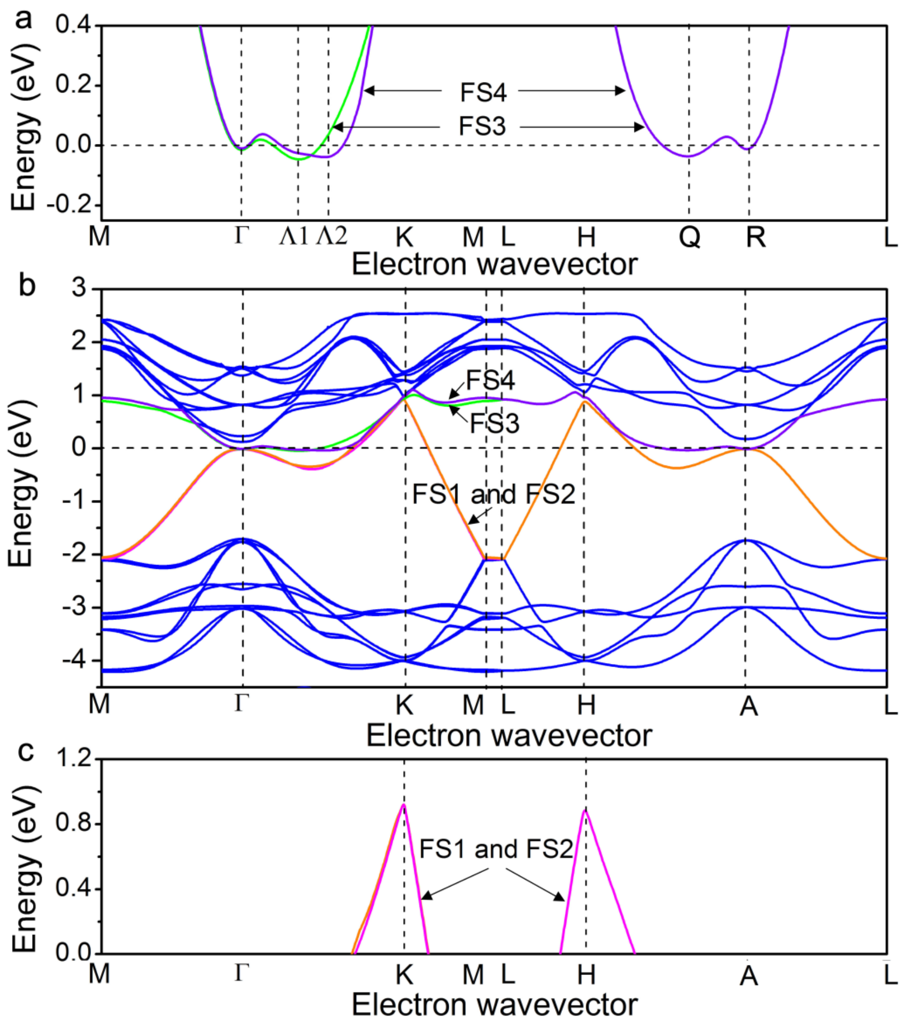

Figure S5. Bands near E_F in Bernal $Ti_3C_2(OH)_2$. (a) Partially degenerated FS3 and FS4, (b) band structure, (c) partially degenerated FS1 and FS2.

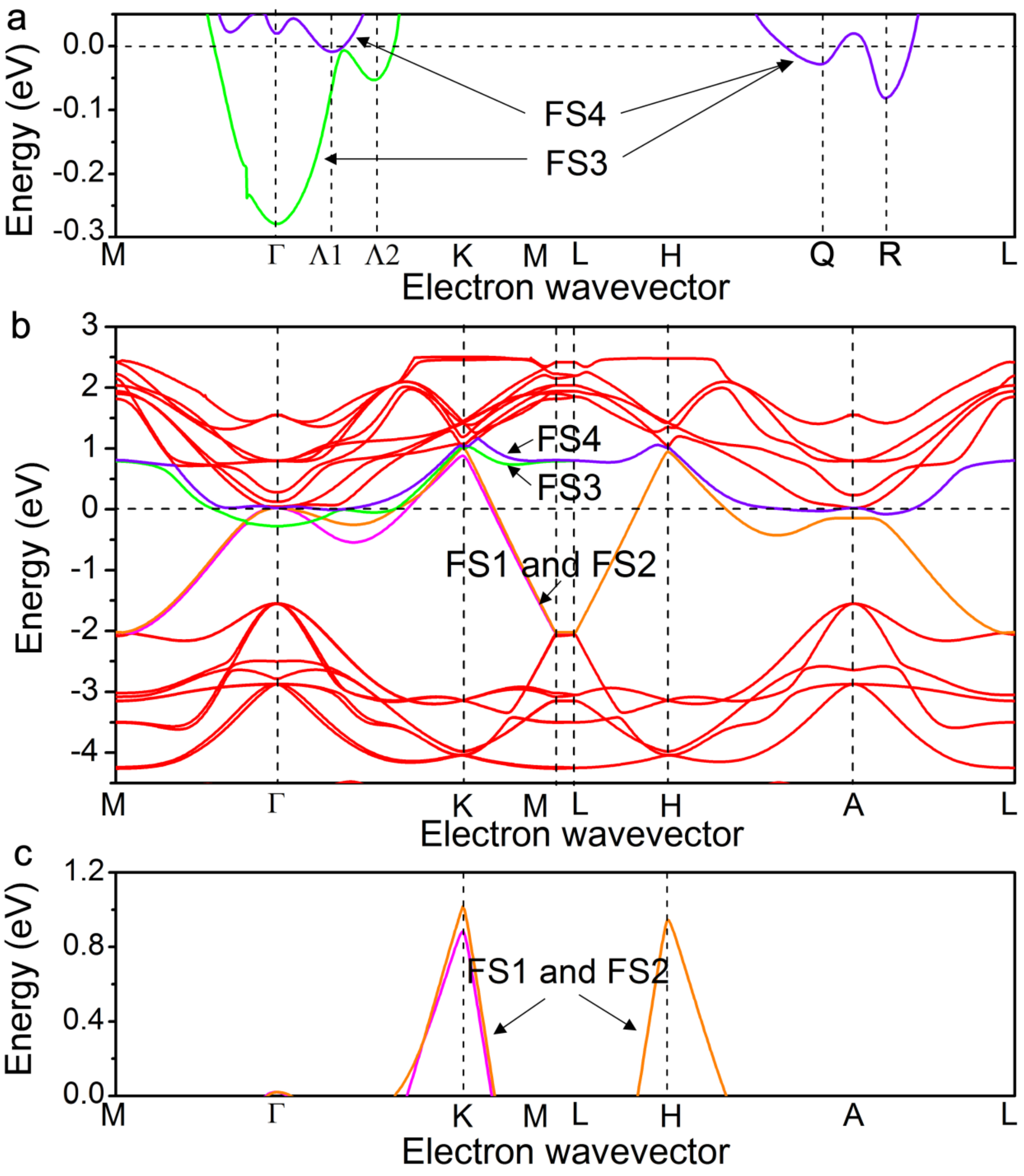

Figure S6. Bands near E_F in SH $Ti_3C_2(OH)_2$. (a) Partially degenerated FS3 and FS4, (b) band structure, and (c) partially degenerated FS1 and FS2.

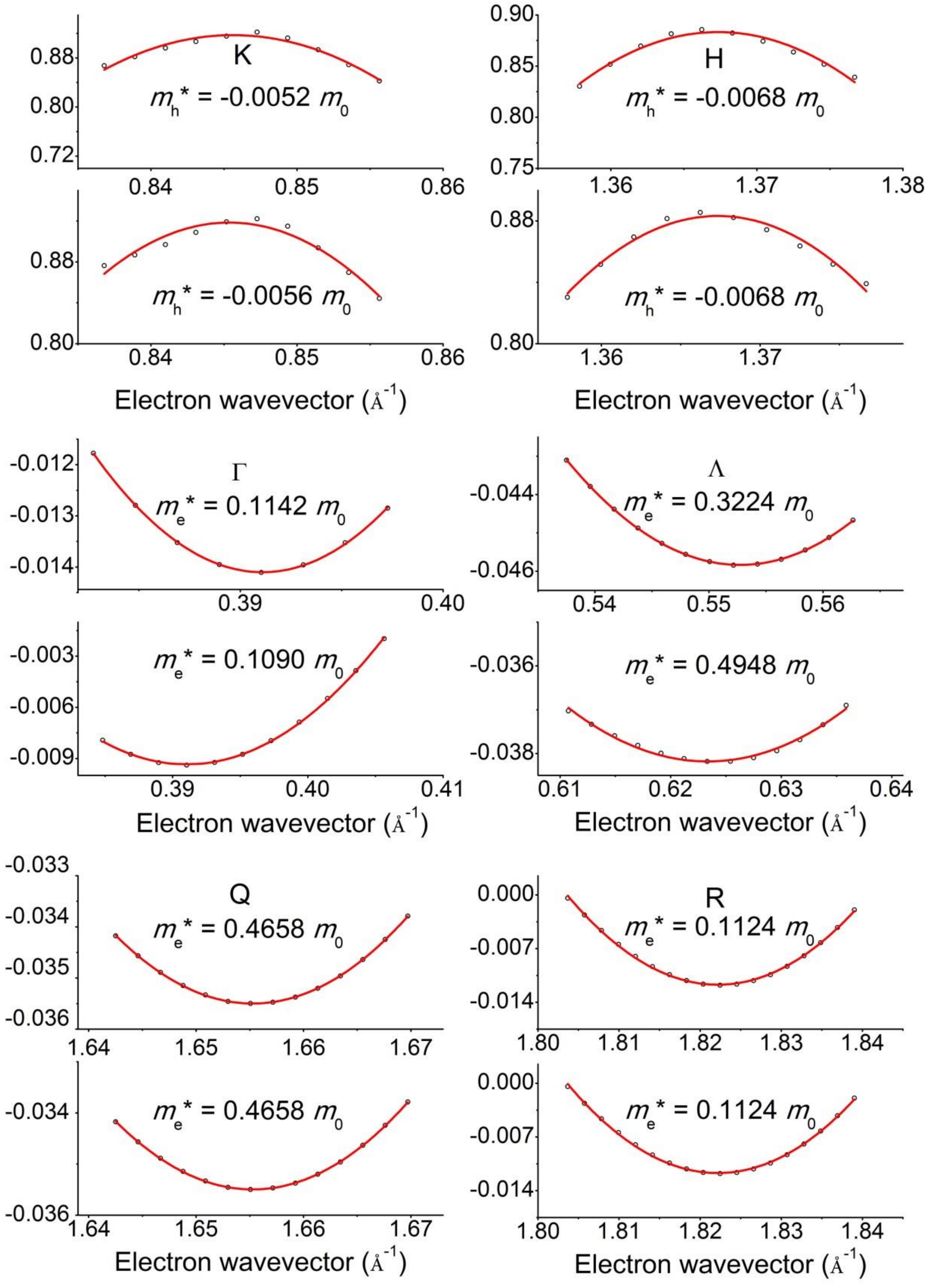

Figure S7. Fitted parabolas for calculating the effective masses of carriers in Bernal $Ti_3C_2(OH)_2$.

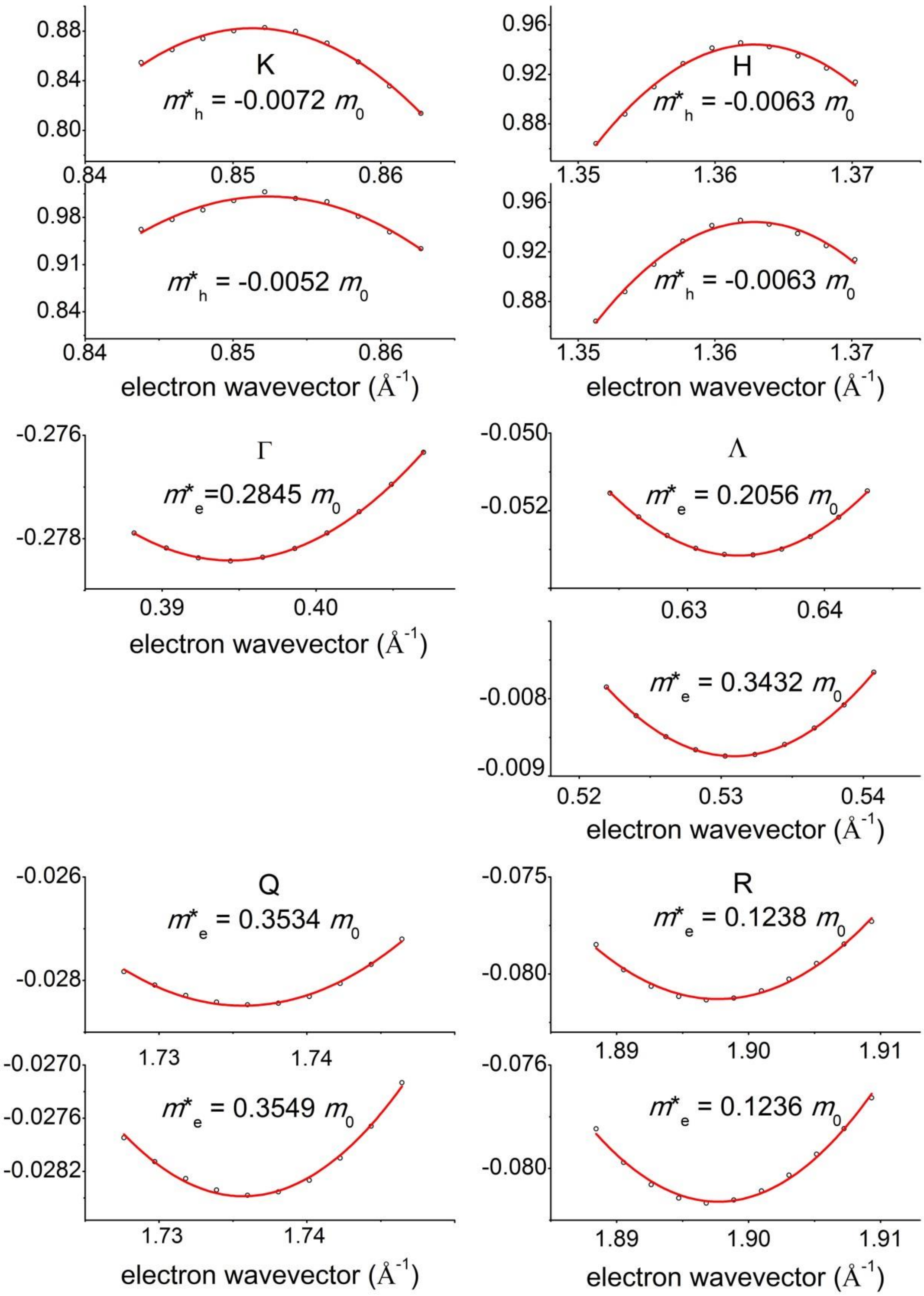

Figure S8. Fitted parabolas for calculating the effective masses of carriers in SH $Ti_3C_2(OH)_2$.

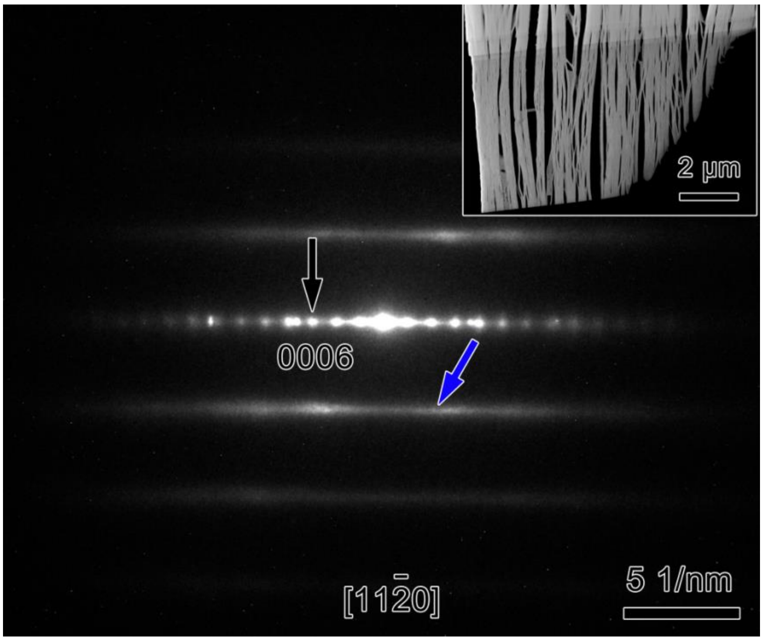

Figure S9. Selected area electron diffraction pattern collected along [11$\bar{2}$0]. Inset is a low-magnification scanning transmission electron microscopy image of the electron transparent $Ti_3C_2T_2$ slab sliced by focused ion beam. The non-periodic combination of different stackings along [0001] results in the strong streaks of (1$\bar{1}$0*l*) diffraction rows (indicated by the blue arrow), which is similar to those of not highly ordered long-period stacking-ordered structure in Mg-Zn/Al-RE (rare earth element) alloys[3–5].

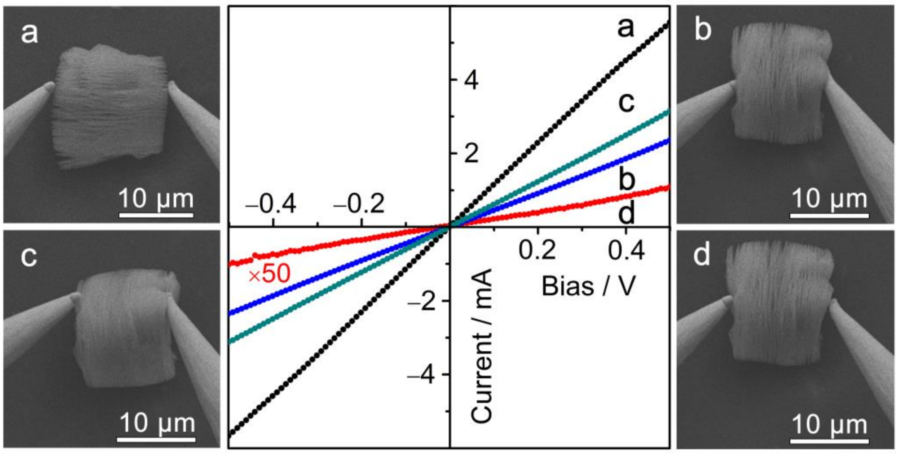

Figure S10. Orientation-dependent *I*−*V* curves and SEM images of an individual accordion-like $Ti_3C_2T_2$ particulate. (a) Along the basal plane. (b) Vertical to the basal plane, and in the modes of (c) shrinking and (d) stretching. The estimated in-plane electrical conductivity is one order of magnitude higher than the vertical electrical conductivity. The electrical conductivity perpendicular to the basal plane measured in shrinking mode is at least 10 times higher than that in stretching mode. For the sake of readability, the vertical axis (current) of curve (d) is scaled, ×50.

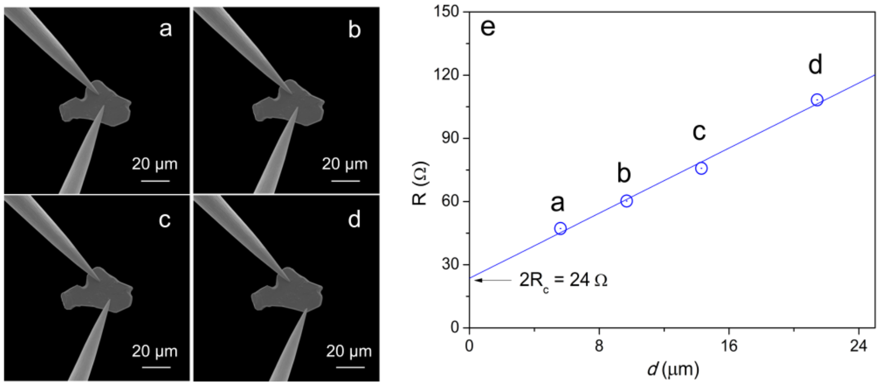

Figure S11. Determination of contact resistance. (**a**–**d**) SEM morphologies of the setups for *I*–*V* measurement. (**e**) Linear fitting of the *R*–*d* curve. As the contact resistance ($\boldsymbol{R}_c$) is part of the total resistance obtained directly from *I*–*V* curves ($\boldsymbol{R}_t$), the intrinsic resistance of the tested materials ($\boldsymbol{R}_i$) is smaller than $\boldsymbol{R}_t$ ($\boldsymbol{R}_t = \boldsymbol{R}_c + \boldsymbol{R}_i$). To evaluate the contact resistance along vertical orientation, a typical crystallite was chosen to measure a series of *I*–*V* curves by increasing the distances between the two probes step by step, as shown in (**a**–**d**). By linearly fitting the *I*–*V* curves, the relationship between $\boldsymbol{R}_c^{\perp}$ and probe distance *d* is established. $\boldsymbol{R}_c^{\perp}$ is estimated to be: $2\boldsymbol{R}_c^{\perp} = 24\ \Omega$ (two probes), which is negligible compared with the vertical resistance (close to 300 Ω, see supplementary Table S4). Since the thickness is very small (< 3 μm), the measurement of $\boldsymbol{R}_c$ between the probe and the side planes ($\boldsymbol{R}_c^{\parallel}$) of the specimen cannot be realized in our laboratory at present. Therefore $\boldsymbol{R}_c^{\parallel}$ is not considered. According to the inequality $\frac{\boldsymbol{R}_i^{\perp}}{\boldsymbol{R}_i^{\parallel}} = \frac{\boldsymbol{R}_i^{\perp}}{\boldsymbol{R}_t^{\parallel} - \boldsymbol{R}_c^{\parallel}} > \frac{\boldsymbol{R}_i^{\perp}}{\boldsymbol{R}_t^{\parallel}}$, using $\boldsymbol{R}_t^{\parallel}$ and $\boldsymbol{R}_i^{\perp}$ to compare the electrical resistivity parallel ($\boldsymbol{R}_t^{\parallel}$, $\boldsymbol{R}_t^{\parallel}$, $\boldsymbol{R}_c^{\parallel}$) and vertical ($\boldsymbol{R}_t^{\perp}$, $\boldsymbol{R}_i^{\perp}$, $\boldsymbol{R}_c^{\perp}$) to the basal plane virtually underestimates the anisotropy. Using the collected data, the electrical resistivity parallel to the basal plane is calculated to be at least 10 times higher than that vertical to the basal plane without considering the $\boldsymbol{R}_c^{\parallel}$. Therefore, albeit the existence of $\boldsymbol{R}_c^{\perp}$ and $\boldsymbol{R}_c^{\parallel}$, the in-plane electrical conduction is much larger (>10 times) than that vertical to the basal plane.

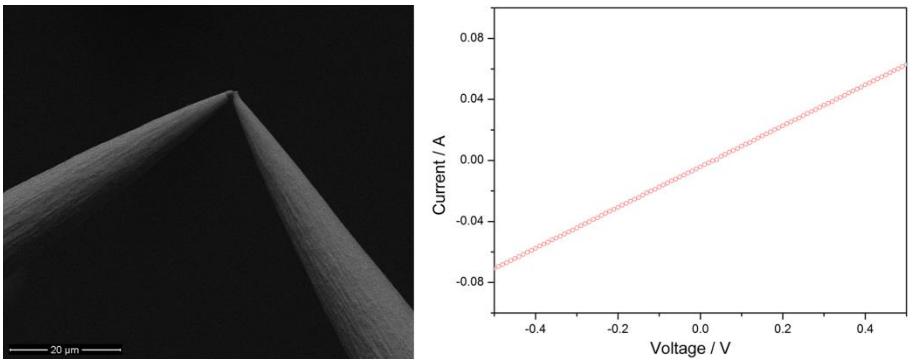

Figure S12. SEM image and *I*−*V* curve. (a) SEM image of the tungsten probes. (b) *I*−*V* curve recorded from two tungsten probes with a tip-tip contact after Joule heating.

1. Zhao, Y. & Spain, I. X-ray diffraction data for graphite to 20 GPa. *Phys. Rev. B* **40**, 993−997 (1989).
2. Coleman, J. N. *et al.* Two-dimensional nanosheets produced by liquid exfoliation of layered materials. *Science* **331**, 568−571 (2011).
3. Yokobayashi, H., Kishida, K., Inui, H., Yamasaki, M. & Kawamura, Y. Enrichment of Gd and Al atoms in the quadruple close packed planes and their in-plane long-range ordering in the long period stacking-ordered phase in the Mg-Al-Gd system. *Acta Mater.* **59**, 7287−7299 (2011).
4. Kishida, K., Yokobayashi, H., Inui, H., Yamasaki, M. & Kawamura, Y. The crystal structure of the LPSO phase of the 14H-type in the Mg-Al-Gd alloy system. *Intermetallics* **31**, 55−64 (2012).
5. Yamasaki, M. *et al.* Highly ordered 10H-type long-period stacking order phase in a Mg-Zn-Y ternary alloy. *Scr. Mater.* **78**−**79**, 13−16 (2014).
